# Taking the biology seriously makes models better

**DOI:** 10.7554/eLife.111070

**Published:** 2026-04-20

**Authors:** Antonio Matas-Gil, Andreas Tiffeau-Mayer

**Affiliations:** 1 Division of Infection and Immunity, University College London London United Kingdom; 2 Institute for the Physics of Living Systems, University College London London United Kingdom

**Keywords:** antibody language model, somatic hypermutation, affinity maturation, mutation-selection model,, antibody engineering, functional prediction, Human

## Abstract

A new biologically-informed training paradigm enables protein language models to predict affinity maturation trajectories for antibodies.

**Related research article** Matsen FA, Dumm W, Sung K, Johnson MM, Rich DH, Starr TN, Song YS, Fukuyama J, Haddox HK. 2026. Separating selection from mutation in antibody language models. *eLife* **15**:RP109644. doi: 10.7554/eLife.109644.

Have you ever wondered how the immune system is capable of repelling most of the pathogens it finds, even if their molecular signatures are completely different to anything it has seen before? Proteins called antibodies that recognize the antigens released by pathogens are key players in this process, as are the B cells that produce them. Antibodies roam the body and can bind to pathogens with specific molecular signatures to neutralize them, or flag them for destruction by other immune cells.

Two important features of the antibody immune system are somatic hypermutation and clonal selection (Figure 1, left). Somatic hypermutation means that the region of an antibody that binds to an antigen experiences very high levels of genetic mutation, which ensures high levels of antibody diversity, while clonal selection favours antibodies with higher affinities for antigens. The phenomenon of B cells producing antibodies with higher and higher affinities over the course of an immune response is known as affinity maturation.

**Figure 1.**
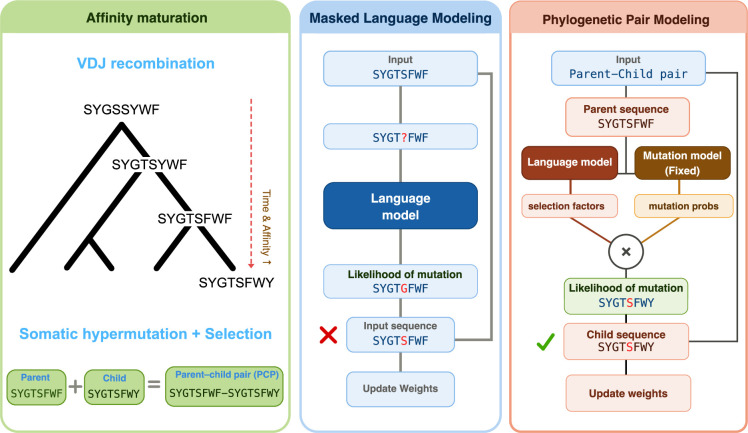
Protein language models and the immune system. Left: Schematic of the process of affinity maturation for antibodies. A process called VDJ recombination generates the initial antibody sequence (SYGSSYWF in this case). Then, during a process called somatic hypermutation, mutations lead to changes in the binding site (here, the second S become a T in the second generation; the Y becomes an F in the third generation; and the final F becomes a Y in the fourth generation). At the same time clonal selection favours antibodies with higher affinities for antigens. This process can be interpreted as an evolutionary tree: the further down we go, the more time has passed and the better the antibody should be. Middle: When using masked language modelling (MLM) to train a protein language model, one of the amino acids in the input sequence is masked (indicated by the question mark), and the model is tasked with finding which amino acid is most likely to fit in the masked location. The prediction of the model is then compared with the actual amino acid, and the weights used in the model are updated accordingly. This approach works well in general, but makes no use of what we know about the biology of antibodies. Right: When using phylogenetic pair modelling to train an antibody language model, the input is a parent–child pair from the antibody evolutionary tree (left), and there are separate language and mutation models. In this approach, the aim is for the language model to learn about clonal selection, as somatic hypermutation can be reasonably well modelled.

Antibodies can also be engineered to act as biological therapeutics. Based on work in the mid-1970s by Georges Köhler and César Milstein (Köhler and Milstein, 1975) – work that led to them sharing a Nobel Prize in 1984 – monoclonal antibodies were first approved by the US Food and Drug Administration in 1986 (Kung et al., 1979; Ortho Multicenter Transplant Study Group, 1985), and their use has grown ever since. Monoclonal antibodies are now the largest class of biopharmaceuticals, helping to treat patients with a range of conditions including cancer, autoimmune disease and various infectious diseases. However, making antibodies with the specificity needed to target a particular disease is challenging, so there is a pressing need for new approaches. Computational protein design has emerged as a tantalizing alternative in recent years, although the complexity of the sequence-structure-function landscape for antibodies means that this approach is also challenging.

Researchers use ‘protein language models’ to design antibodies. These models are similar to the large language models used by platforms such as ChatGPT, but they work with sequences of amino acids instead of natural language. Protein language models are trained by masking an amino acid in an existing protein, and asking the model to guess the identity of the amino acid that was masked. This training strategy, which is called masked language modelling (MLM), allows the model to learn general features of protein biology (Figure 1, middle). This approach has seen success in areas such as protein structure prediction, but it has not been as successful when applied to other problems, such as protein function prediction (Li et al., 2024). Nevertheless, MLM training remains the dominant training paradigm.

Now, in eLife, Frederick Matsen of the Fred Hutchinson Cancer Center and colleagues report a new training strategy that is specifically designed for antibodies (Matsen et al., 2026). This strategy takes advantage of the fact that the mutations initiated by an enzyme called AID (short for activation-induced cytidine deaminase) during somatic hypermutation exhibit sequence preferences that arise from the mutational mechanism itself rather than from clonal selection (Figure 1, right). In short, the AID enzyme deaminates a cytosine base in DNA, turning it into uracil, which is then processed by DNA repair pathways such as base excision and mismatch repair. Together these processes result in characteristic, non-random mutation patterns, which can be inferred from data.

Matsen et al. first make the limitation of current approaches explicit by showing that MLM-trained protein language models essentially learn the biased sequences introduced by somatic hypermutation, even though these sequences do not directly reflect functional selection pressures. Having identified this issue, the researchers then propose an ingenious solution rooted deeply in the biology of antibodies and the affinity maturation process.

The new approach, which we propose calling phylogenetic pair modelling, allows Matsen et al. to train a deep amino acid selection model (DASM). Importantly, phylogenetic pair modelling separates the contributions of mutation and selection, extending previous work in the field by themselves (Matsen et al., 2025) and others (Elhanati et al., 2015). Training involves working with pairs of sequences – a parent sequence, and the child sequence after somatic hypermutation – and adjusting the parameters of the DASM to optimise the predictions of the model. When adjusting parameters, existing models of somatic hypermutations are used to account for the underlying probabilities of mutations, thus focusing the DASM on learning the selection pressures.

The researchers find that using phylogenetic pair modelling to train their model allows it to substantially outperform existing models of larger size. The beauty of this new approach to training is that its improvement in performance is not driven by extra computational training, which is costly, but by a conceptually simple, biologically-informed training procedure. The end result is a model that is both smaller and faster than existing models.

Looking forward, approaches such as those described here will be crucial for understanding the fundamental biology of the affinity maturation process, as well as for the development of antibody-based therapies. More broadly, the work of Matsen et al. showcases the importance of training strategies, as has also been demonstrated for another component of the immune system – T cell receptors: last year it was shown that a training method called contrastive learning can help protein language models in this field to perform better by counteracting recombination biases (Nagano et al., 2025). Collectively, these strategies demonstrate how true interdisciplinarity – a combination of biological domain knowledge and machine learning know-how in this case – can tackle complex scientific challenges.
